# Nailing treatment in bone transport complications

**DOI:** 10.1007/s11751-014-0196-9

**Published:** 2014-07-24

**Authors:** C. Biz, C. Iacobellis

**Affiliations:** Orthopaedic Clinic, Department of Surgery, Oncology and Gastroenterology DiSCOG, University of Padua, Via Giustiniani 2, 35128 Padua, Italy

**Keywords:** Bone transport, Distraction osteogenesis, External fixator, Intramedullary nailing

## Abstract

A series of cases of reamed intramedullary nailings carried out after complications in regenerated bone and docking site had occurred in bone transport is presented here. Nine patients (femur = 5; tibia = 4) had treatment with resection after open fractures or infection and underwent bone transport. The mean length of regenerated bone was 9.5 cm (range 6–18 cm). After bone transport, the fixator remained in place for a mean period of 12.8 months (range 8–24 months). In six cases (femur 4; tibia 2), the thickness of the cortical wall of the regenerate column was insufficient, and in two of these, there was, in addition, nonunion of the docking site. In the two tibial cases, nailing was carried out shortly after the fixator had been removed and after refracture of the regenerated bone had occurred due to insufficient cortical thickness. In one femur, nailing was carried out for nonunion of the docking site. Follow-up involved clinical and X-ray checks. The mean follow-up was 3.9 years (range 2–6 years). In all cases, union and with complete corticalization of the regenerate column was observed at an average 6 months after nailing (range 4–11 months). Infection occurred in one tibia 4 months after nailing. The infection was treated with antibiotics, and the nail was subsequently removed. We conclude that nailing is a potential solution for regenerated bone and docking site problems but, if used after prolonged periods of external fixation, may necessitate antibiotic therapy for at least 10 days after the fixator has been removed.

## Introduction

Bone transport for segmental resections in the treatment for infected nonunion, osteomyelitis, or after bone loss in open fractures remains a major undertaking for orthopedic surgeons [1–4]. For long-bone diaphyseal defects larger than 5 cm, with or without a soft-tissue defect, specialized management is needed [5]. The use of vascularized bone grafts [6, 7], allograft bone transplantation, or bone transport by an external fixator alone or over an intramedullary nail has been reported in [8–10]. Bone transport with a circular or monolateral external fixator represents a standard method for managing lower limb bone defects and for limb lengthening [11–14]. These methods induce two biological processes: distraction osteogenesis, the new production of bone from a corticotomy, and transformational osteogenesis, where the mechanical stimulation of an abnormal bony interface regenerates normal bony continuity and achieves consolidation [15, 16]. Further, the regenerated bone formed by bone transport is mechanically stronger to that formed by bone grafting but there is a risk of refracture after frame removal [17]. Distraction osteogenesis by the Ilizarov technique [18–20], subsequently modified by Cattaneo et al. [21], has been used successfully in all long bones since its introduction [17, 22–26]. In contrast, bone transport using a monolateral external fixator achieves a similar result through distraction of callus (callotasis) that is obtained from a subperiosteal osteotomy [27]. Compared with a circular frame, this device has the advantage of being lighter and a simpler application. There is also less soft-tissue transfixation by pins, thereby allowing early physical exercise and partial weight-bearing [28]. Use of hydroxyapatite-coated pins decreases pin site-related problems [9, 29].

Bone transport carries advantages of minimal soft-tissue trauma, almost limitless reconstruction of bone defects and elimination of donor site morbidity [30, 31]. The process of bone transport using an external fixator alone is still a lengthy and uncomfortable process. It is a labor-intensive surgical procedure and subjects to many complications with considerable treatment times [32–36]. Despite the versatility of distraction osteogenesis, both patients and orthopedic surgeon are prompted to remove the external fixator early to decrease discomfort and complications [37] of which the most frequent are nonunion at the docking site [38], fracture of regenerated bone due to the lack of internal stabilization, failure of distraction osteogenesis, and recurring infection [39, 40]. Simpson and Kenwright [41] report a fracture rate of 9.4 % in a series of 180 lengthening segments; O’Carrigan [42] reports an 8 % fracture rate in 650 patients with 986 lengthening segments, and Danziger [43] had refracture of the femur in 6 of 18 patients. Lavini [38] had axial deviation in 17.6 % in a series of 17 cases. In our previous study of 100 consecutive cases of bone transport using the Ilizarov method, we found 1 % refracture of the newly formed bone segment of the tibia, 17 % nonunion at the docking site in 10 femurs and 7 tibias, 10 % bone transport arrest due to the failure of distraction osteogenesis in 2 femurs and 8 tibias, and 4 % of cases had recurring infection [22]. Furthermore, paresthesiae (9 %) [32, 44], angulation, and deformity of the newly formed bone column (2–17 %) [22, 38, 44], and neighboring joint contractures due to increased soft-tissue tension and joint stiffness (10–28 %) [22, 44], are encountered frequently during lengthening and bone transport.

One solution to some of these complications is the insertion of an intramedullary nail in order to support the regenerated bone during the consolidation phase and facilitate the removal of the external fixator after the distraction phase of lengthening. However, intramedullary nailing after bone transport with a circular or monolateral external fixator is still controversial as there is a risk of infection. Specifically, when combining external and internal fixation, the risk of deep infection has been reported between 3 and 15 % [45, 46]. Other methods employed to shorten the external fixation treatment period have been described: docking site stimulation with autogenous bone graft, bone marrow injection, electric or magnetic field stimulation, ultrasound stimulation, and the use of bone growth stimulating factors [47].

This retrospective study was carried out on a sample of patients treated with reamed intramedullary nailing after bone transport with the aim of assessing the evolution of union and the incidence of infection and major complications after surgery.

## Materials and methods

This is a retrospective review of a case series. All subjects participating in this study were counselled over the risks and benefits of the procedure; informed consent for inclusion in this retrospective case series was obtained. Between 2006 and 2010, nine patients (eight males, one female; average age 35.5 years; range 25–57) underwent bone transport for bone loss after open fractures and infection. All patients had bone defects of >5 cm after resection and debridement. There were no specific exclusion criteria. Five femurs and four tibias were involved. In all cases, samples were taken for culture. These included no fewer than four swabs both before and during surgery. The cause of infection was identified as *Staphylococcus aureus* in six cases and *Pseudomonas aeruginosa* in 3. Subsequent antibiotic therapy was carried out, according to the culture and sensitivity results, for a minimum 6-week period or until the erythrocyte sedimentation rate and C-reactive protein level had returned to normal [48].

**Fig. 1:**
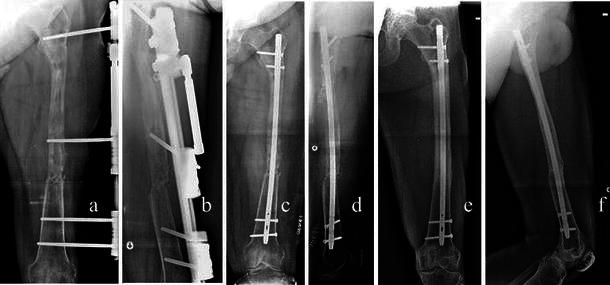
Case 2: **a**, **b** preoperative X-ray, **c**, **d** postoperative checkup, **e**, **f** follow-up 5 years later

**Fig. 2:**
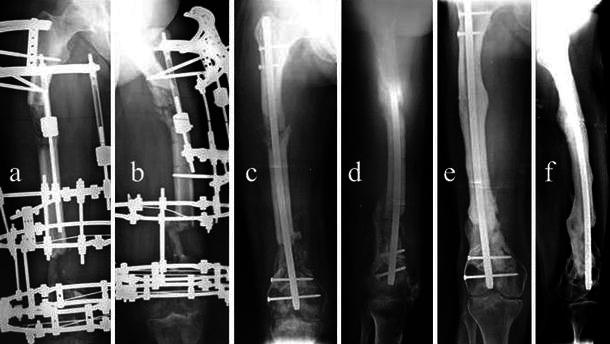
Case 3: **a**, **b** preoperative X-ray; bony bridge in the docking site, arrest of transport, varus, and procurvatus of the regenerated bone, **c**, **d** follow-up 40 days later, **e**, **f** follow-up 4 years later

Six patients were treated using the Ilizarov circular fixator (Amplimedical s.p.a, Milan, Italy) and 3 with a monolateral rail fixator (Limb Reconstruction System Orthofix SRL, Verona, Italy). The types of transport through healthy tissue were descending (proximal–distal) in two cases (femurs), ascending in four cases (one femur, three tibias), double transport in two femurs (with mid-diaphyseal contact of transported bone ends at the docking site from proximal and distal metaphyseal osteotomies), and in one tibia (twin transport from a double proximal osteotomy). The mean length of regenerated bone was 9.5 cm (range 6–18). At the end of transport, the fixator was kept in place for a mean period of 12.8 months (range 8–24). In all cases, the docking site was exposed, the interposed tissue was removed, and if small residual gaps were seen between the two bone ends, cancellous bone was taken from the ipsilateral iliac crest and grafted. We observed spontaneous healing of the skin defects at the docking site; plastic surgical cover was not required. Reaming and nailing (Synthes nail) were carried out in six cases (four femurs, two tibias; cases 1–4, 6, 9) for which the thickness of the cortical wall of the regenerated bone was deemed insufficient (Fig. 1) and removal of the fixator would have created a high risk of fracture. In two of these six patients (cases 4 and 9), nonunion of the docking site was diagnosed additionally. In another patient (case 3; Fig. 2), a bony bridge had formed between the intended docking site and transport segment causing an arrest of transport and varus and procurvatum deformity of the regenerated bone. For this case, as well as intramedullary nailing, the docking site was filled with autogenous bone grafts. In two patients (cases 5 and 7, both tibial defects), nailing was carried out for refracture of regenerated bone, which occurred soon after fixator removal. In one patient (case 8, femoral defect), intramedullary nailing was carried out for nonunion of the docking site. In all cases, the fixator was removed prior to nailing and a plaster cast was applied for 10 days. Details of the cases are summarized in Table 1.

**Table 1:** Cases

Case	Gender [age (years)]	Bone, side (L, left; R, right)	Open fracture Gustilo I, II, III	Findings on culture	Bone loss (cm)	Fixator	Transport: A, ascending technique. D, descending T. DL, double-level bone T	External fixator time (months)	Regenerated bone* IC, R	Docking site* C, N
1	M, 32	Femur, R	II	*Staphylococcus aureus*	10	Orthofix	DL	11	IC	C
2	M, 28	Femur, L	I	*Pseudomonas aeruginosa*	6	Orthofix	D	12	IC	C
3	M, 47	Femur, R	II	*Staphylococcus aureus*	9	Ilizarov	D	8	IC	N
4	M, 28	Femur, L	II	*Staphylococcus aureus*	14	Orthofix	DL	11	IC	N
5	M, 57	Tibia, L	I	*Staphylococcus aureus*	6	Ilizarov	A	9	R	C
6	F, 25	Tibia, L	II	*Staphylococcus aureus*	18	Ilizarov	A	24	IC	C
7	M, 44	Tibia, L	II	*Pseudomonas aeruginosa*	7	Ilizarov	DL	10	R	C
8	M, 28	Femur, L	I	*Pseudomonas aeruginosa*	6	Ilizarov	A	13	C	N
9	M, 31	Tibia, L	II	*Staphylococcus aureus*	10	Ilizarov	A	18	IC	N

*C Consolidation, *N* nonunion, *IC* insufficient corticalization, *R* refracture

Patients were followed up at 2-month intervals until X-rays showed corticalization with bone thickness equal to that of the bone adjacent to the regenerated bone and/or consolidation of the docking site. The functional outcome measures were recorded, they are as follows: an observable limp, stiffness of the principal joints (defined as >70° loss of knee flexion or >15° loss of knee extension, >50° loss of ankle motion, all as compared with the normal contralateral side), and the ability to fully weight-bearing pain-free. The limb and bone segment was assessed radiologically for axial deformity, union, and for signs of infection after nailing. Fractures after nail removal were noted. The outcome was considered excellent if the patients were fully weight-bearing, pain-free, without knee and ankle stiffness, and had a normal aligned limb without need for further surgery after the intramedullary nailing had been performed; good if the patients required more surgery to achieve union; and poor if major complications occurred according to Paley’s classification [33]. The patients were asked whether they were satisfied with the procedure or would have preferred primary amputation instead of the multiple procedures undertaken to salvage the limb. No statistical analysis was performed as the number of the cases is small.

## Results

**Fig. 3:**
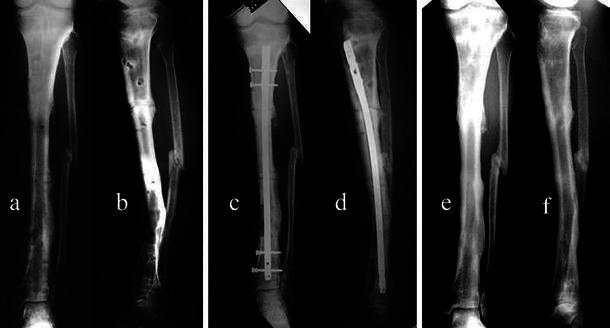
Case 5: **a**, **b** preoperative X-ray, 1 month after the removal of the fixator, **c**, **d** postoperative checkup, **e**, **f** follow-up 3 years later

The mean follow-up was 3.9 years (range 2–6). All cases had undergone resection and bone transport for open fractures after road traffic accidents. Complete corticalization of the regenerate column of bone was achieved on average after 6.5 months (range 4–11) after nailing. The length of regenerated bone was checked before and after nailing, and in no case was shortening of regenerated bone observed. None had major complications, neurovascular injuries, joint subluxations or fracture of the regenerated bone. Using the criteria described earlier, eight patients obtained excellent results and only one patient a good result as further surgery was needed; in this case, infection of the tibia occurred 4 months after nailing despite corticalization (case 5, Fig. 3). The nail was removed and the infection treated. The patient was re-examined 3 years after nail removal and was found to be without signs of recurrence. In three other cases, the nail was removed at the patient’s request. One patient was found to have knee stiffness that did not require further surgery. All of the patients were satisfied with the procedure, and none expressed a preference for amputation despite the multiple procedures or length of treatment. The patient outcome data are summarized in Table 2.

**Table 2:** Results

Case	Follow-up (years) after nailing	Limp	Knee (K) or ankle (A) stiffness	Fully W–B and pain-free	Axial deviation	Infection after nailing	Consolidation after nailing (months)	Removal of the nail	Fractures after nail removal
1	4	No	No K–A	Yes	No	No	10	No	–
2	5	No	No K–A	Yes	No	No	11	No	–
3	4	Yes	Yes K; No A	No	No	No	6	No	–
4	3	No	No K–A	Yes	No	No	8	No	–
5	3	No	No K–A	Yes	No	Yes	4	Yes, after 4 months	No
6	5	No	No K–A	Yes	No	No	6	Yes, after 6 months	No
7	6	No	No K-A	Yes	No	No	4	Yes, after 5 years	No
8	3.5	No	No K–A	Yes	No	No	4	Yes, after 3.5 years	No
9	2	No	No K–A	Yes	no	No	6	No	–

## Discussion

Several reports in the literature show good results from nailing after or during external fixation. Femoral and tibial nailing with reaming is used commonly after damage control stabilization with external fixation for cases of multiple trauma [16] or open fractures of Gustilo type III [49, 50]. After the removal of the fixator, intramedullary nailing is carried out in the same operating session or delayed to occur after a period of traction [16, 49] or time in plaster [49, 50], (with or without an interim period of antibiotic therapy) for fear that the pins and wire sites could lead to potential deep intramedullary infections. As early as 1956, Bost et al. [51] described a lengthening technique with an inserted nail, involving external devices and Steinmann pins applied to the same bone segment. Forty years later, Paley et al. [52] presented a series of 32 cases of femoral lengthening using external fixators (Ilizarov or Orthofix) over intramedullary nails ensuring the pins or wires did not contact the nail. At the end of the lengthening period, the fixator was removed and the nail locked. Other cases of lengthening where external fixators were combined with intramedullary nails have also been reported [53–55]. In 39 cases of lengthening, Rozbruch et al. [53] reported one deep infection that was treated by nail removal. The authors attributed the low number of infections partly to the fact that the regenerated bone was well vascularized. In 13 lengthenings with fixators and nailing, Bilen et al. [54] had no cases of infection. In 56 lengthenings with fixators and nails which were either unreamed or only very slightly reamed, Park et al. [55] reported no deep infections and only 13 pin track infections, all which resolved with antibiotics. The literature also contains several reports describing intramedullary nails in bony segments already partially resected due to previous infections. Papineau [56] inserted a nail 2 weeks after surgical debridement and later filled the gap with cancellous bone grafts. Several other authors [57–60] have presented cases of bone transport with fixators and nailing. Raschke et al. [57] have adopted a more cautious approach; in four cases of open tibial fractures of Gustilo types II and III, they first proceeded with debridement and application of external fixators, and after 4–6 weeks inserted locked undreamed intramedullary nails in combination with new monolateral fixators. There were no infections of the medullary canal. In 2002, Lai et al. [15] presented 27 cases of bony transport in femurs and tibias with regenerated bone or docking site problems or refractures. In these cases, with an average of 3–4 weeks after the removal of the fixator, reamed locked nails were inserted. Two cases of infection at the site of the distal docking screws were resolved after nail removal. Eralp et al. [58] presented a series of 17 resections due to chronic osteomyelitis, with antibiotic therapy for 6 weeks and then bone transport with reamed nails and fixators concurrently. They reported three deep infections. In another series of 17 patients, Li et al. [59] used external fixators with reamed nails for bone transport to resolve large defects of the femur which were created after resection for osteomyelitis; the patients were subjected to a 6-week course of antibiotic therapy before bone transport surgery was performed. They reported 10 superficial pin track infections and one deep intramedullary infection that was treated by the removal of the nail and external fixator and reaming of the medullary canal.

These reports suggest that a nail may be inserted (despite previously infected tissue) if there is interim antibiotic therapy. In our series where bone transport was performed after resection for infection, we inserted the intramedullary nails after an interval of antibiotic therapy. We had one case (case 5) of re-infection after nailing, which we resolved by nail removal after union and antibiotic treatment (Fig. 3). Although some authors [60] believe that the risk of expanding an infection into the medullary cavity increases with the insertion of an intramedullary nail, it is our belief this risk is reduced if the previous site of infection is thoroughly debrided and some time allowed to pass before nailing. This is in order to clear up pin infections that often occur, particularly along screws. Kirschner wires, having a smaller diameter, create less serious infections than those produced by pins. Once the infection is treated and resolved, the nail can be inserted after reaming, which serves as a biological stimulator for the corticalization of the regenerate area. Intramedullary reaming for chronic osteomyelitis results may assist in removing the laminar endosteal sequestra of the tibial canal as well as diminishing the intraosseous pressure; the bone is revascularized through an improved periosteal circulation [61–63]. Several authors have also reported their experience with reaming [64–68] showing that reamed bone has considerable osteoblastic potential, equal to that of the iliac crest [66]. Frölke et al. [66] and Wenisch et al. [67] report that human reaming debris is a source of multipotent stem cells that can grow and proliferate in vitro. In a recent review, Brinker et al. [68] stated that in the cases of nonunion, insertion of a second nail after the first promotes healing as long as the canal is reamed again and a larger nail inserted. These considerations may explain the corticalization effect that we found in our cases.

Bone transport is a reliable method for the reconstruction of bone defects in femur and tibia, and remains a safe treatment dealing with defects after resection for bone infection. Similarly, nailing is a good solution for regenerated bone and docking site problems as long as antibiotic therapy is prescribed, and nailing is carried out at least 10 days after the fixator has been removed. Complications due to deep infections are not common and may be resolved.

There are weaknesses in this case series. We acknowledge the small number of the patients and a potential bias due to its retrospective design being major limitations. The literature is limited on the subject of nailing treatment in bone transport complications. This report adds some support to a successful alternative strategy for the treatment for complications of bone transport with a moderate-term follow-up.
